# Deep Brain Stimulation Impedance Decreases Over Time Even When Stimulation Settings Are Held Constant

**DOI:** 10.3389/fnhum.2020.584005

**Published:** 2020-11-09

**Authors:** David Satzer, Huiyan Yu, Meredith Wells, Mahesh Padmanaban, Matthew R. Burns, Peter C. Warnke, Tao Xie

**Affiliations:** ^1^Department of Neurosurgery, University of Chicago Medicine, Chicago, IL, United States; ^2^Department of Neurology, Beijing Hospital, National Center of Gerontology, Beijing, China; ^3^Department of Neurology, University of Chicago Medicine, Chicago, IL, United States; ^4^Department of Neurobiology, University of Chicago Medicine, Chicago, IL, United States; ^5^Department of Neurology, University of Florida College of Medicine, Chicago, IL, United States

**Keywords:** impedance, current, voltage, DBS, STN, Parkinson’s disease, VIM, tremor

## Abstract

**Objectives**: To study whether and to what extent the therapeutic impedance and current change under long-term deep brain stimulation (DBS) with constant stimulation settings, which could inform the role of constant current stimulation.

**Methods**: Therapy impedance and current measurements were retrospectively collected from patients with Parkinson’s disease (PD) undergoing DBS of the subthalamic nucleus (STN) or essential tremor (ET) undergoing ventral intermediate nucleus (VIM). Baseline and follow-up measurements were obtained for intervals of at least 6 months without changes in stimulation settings. The single longest interval of constant stimulation for each electrode was included. Temporal trends in impedance and current were analyzed as absolute and relative differences and as the rate of change.

**Results**: Impedance and current data from 79 electrodes (60 in STN, 19 in VIM) in 44 patients (32 with PD, 12 with ET) met inclusion criteria. The duration between baseline and follow-up measurements with constant stimulation settings was 17 months (median, with an interquartile range of 12–26 months) in the mixed group. Therapy impedance decreased by 27 ± 12 Ω/year (mean ± 2 standard errors; *p* < 0.0001), and therapy current increased at a rate of 0.142 ± 0.063 mA/year (*p* < 0.0001). Similar results were observed in the STN and VIM subgroups.

**Conclusions**: Impedance decreases gradually over time, even when stimulation settings are kept constant. The rate of decrease is smaller than previously reported, suggesting that changes in stimulation settings contribute to impedance drift. Stimulation-independent impedance drift is gradual but relevant to constant-current programming.

## Introduction

Deep brain stimulation (DBS) has proven to be of significant therapeutic benefits for patients with Parkinson’s disease (PD), essential tremor (ET), and dystonia (Vidailhet et al., 2005; Weaver et al., 2009; Follett et al., 2010; Williams et al., 2010; Deuschl et al., 2011). DBS has traditionally relied on constant-voltage therapy, in which the stimulation voltage is set and current delivery varies according to electrical impedance. However, constant current therapy has become increasingly used to provide more stable energy delivery (Okun et al., 2012). One of the major reasons for this is the downward drift in impedance over time (Satzer et al., 2014; Hartmann et al., 2015; Wong et al., 2018). The cause of this drift is unknown; gradual accumulation of cerebrospinal fluid around the electrode has been proposed as a potential mechanism (Satzer et al., 2014). Many factors are known to affect impedance; these include stimulation voltage, contact activity, target nucleus, and contact location concerning target nucleus (Cheung et al., 2013; Satzer et al., 2014, 2015; Wong et al., 2018). Electrode position is fixed after implantation, but stimulation settings are frequently adjusted due to disease progression and evolving patient needs. Impedance has been reported to immediately decrease after contact activation and higher stimulation voltages have been associated with lower impedance values (Satzer et al., 2014). While prior studies have employed multivariate analysis of impedance changes over time, stimulation settings have not been held constant in any of these studies. Since stimulation is associated with lower impedance, it is conceivable that a long-term increase in programmed energy delivery (compensating for disease progression) could entirely account for the observed downward impedance drift over time.

This study aimed to assess if impedance declines over time when stimulation settings are kept unchanged. Impedance has been associated with clinical response to DBS (Satzer et al., 2015). Changes in impedance over time may affect long-term benefits and motivate constant-current stimulation. Additionally, the presence or absence of stimulation-independent impedance drift can expand the understanding of the brain-electrode interface.

## Materials and Methods

### Study Design and Participants

This retrospective study was approved by the Institutional Review Board at the University of Chicago Medicine. All patients included in this study underwent DBS electrode placement (Medtronic, MN, USA) in the bilateral or unilateral subthalamic nucleus (STN) for PD or ventral intermediate nucleus of the thalamus (VIM) for ET. Patients with an interval of at least 6 months without changes in stimulation settings (voltage, pulse width, frequency, and active contact) between 2016 and 2019 were identified. Only patients undergoing constant-voltage monopolar stimulation were included. Data from the first 6 months following electrode implantation were excluded due to known early post-operative impedance fluctuation (Lempka et al., 2009). Stimulation settings, including therapy impedance (i.e., impedance measured from active contacts at the therapeutic stimulation settings) and current, were recorded from the initial and follow-up visits. Only one interval with stable stimulation settings was included per electrode; in the case of multiple such intervals, the longest interval was selected. In patients with bilateral DBS electrodes, each electrode was treated independently.

### Outcome Measures and Predictors

Therapy impedance (Ohms, Ω) and current (milliampere, mA) were measured at the baseline visit (i.e., visit at the start of > 6-month interval) and follow-up visit (i.e., visit at end of >6-month interval). Time since baseline visit, DBS target (STN or VIM), and electrode laterality (left or right) were recorded. Patient demographics and fixed stimulation parameters were recorded as well.

### Statistical Analysis

Data for several variables diverged from a normal distribution, and nonparametric statistical analysis was used unless otherwise specified. Subgroup composition by sex was compared between STN and VIM subgroups with the chi-squared test. Other demographic data and stimulation parameters were compared between STN and VIM subgroups with the Mann–Whitney-*U* test.

Relative change in impedance and current was calculated as value at follow-up minus value at baseline, divided by baseline value. Absolute and relative changes were compared to a hypothetical mean of zero with the Wilcoxon rank-sum test.

The rate of change of impedance and current over time was assessed with a mixed linear regression model. Impedance or current was used as the dependent variable; time, target, and laterality were analyzed as fixed effects; and a random effect for electrode was introduced to account for variation between electrodes.

An alpha level of 0.05 was used for all significance testing. Statistical analysis was performed with SAS University Edition (SAS Institute Inc., Cary, NC, USA).

## Results

### Patient Characteristics

Forty-four patients (32 with PD, 12 with ET) and 79 DBS electrodes (60 in STN, 19 in VIM) met the study criteria. Demographic information and stimulation parameters are reported in Table 1. The median length of the longest interval with constant stimulation parameters was 17 months (interquartile range of 12–26 months). There was no difference between the STN and VIM subgroups in the sex distribution, age at disease onset, age at DBS placement, length of study interval, voltage, or pulse width. The frequency was lower for STN electrodes (median 130 Hz, interquartile range 60–130 Hz) than VIM electrodes (median 130 Hz, interquartile range 130–180 Hz; *p* = 0.004), since 17 of 60 STN electrodes were programmed at 60 Hz whereas no VIM electrodes were programmed at low frequency, and several VIM electrodes were programmed as high as 180 Hz.

**Table 1:** Patient demographics and stimulation settings.

	STN+VIM	STN	VIM	p (STN vs. VIM)
Patients/electrodes	44/79	32/60	12/19	-
Female^1^	13	10	3	0.73
Age at disease onset^1^ (years)	54 (45–57)	54 (45–57)	48 (19–59)	0.37
Age at DBS placement^1^ (years)	63 (58–69)	63 (58–69)	62 (57–71)	0.82
Longest interval without change in stimulation settings^1^ (months)	17 (12–26)	17 (12–26)	11 (7–25)	0.28
Amplitude^2^ (V)	3.0 (2.2–3.4)	3.0 (2.2–3.3)	3.1 (2.3–3.8)	0.22
Frequency^2^ (Hz)	130 (130–130)	130 (60–130)	130 (130–180)	0.004
Pulse width^2^ (μs)	60 (60–60)	60 (60–60)	60 (60–90)	0.08

*Values are expressed either as counts or as median (interquartile range). ^1^Statistics by the patient. ^2^Statistics by the electrode. V, volts. Hz, Hertz. μs, microseconds*.

### Changes in Impedance and Current

**Figure 1:**
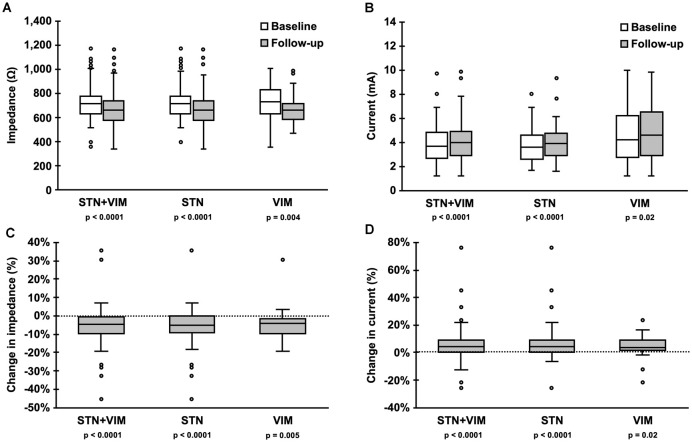
Changes in impedance and current over the study period. Box-and-whisker plots of **(A)** impedance and **(B)** current at baseline (white) and follow-up (gray) measurements show a significant decrease in impedance, and a commensurate increase in current, for the entire cohort as well as the subthalamic nucleus (STN) and ventral intermediate nucleus (VIM) subgroups. Likewise, box-and-whisker plots of percent change in panel **(C)** impedance and **(D)** current show a significant decrease in impedance and increase in current for the entire cohort and the STN and VIM subgroups.

Significant absolute and relative reduction in the impedance was found in the entire cohort (*p* < 0.0001) and in the STN (*p* < 0.0001) and VIM (*p* < 0.01) subgroups (Table 2; Figure 1). Significant increase in the absolute value and percentage change of current were also found in the entire cohort (*p* < 0.001) and in the STN (*p* < 0.001) and VIM (*p* = 0.02) subgroups (Table 2; Figure 1).

**Table 2:** Changes in impedance and current over the study period.

		STN+VIM	STN	VIM
Therapy				
Impedance (Ω)	Baseline	719 (627–780)	719 (627–774)	727 (630–827)
	Follow-up	658 (575–738)	661 (574–739)	658 (582–718)
	p (baseline vs. follow-up)	<0.0001	<0.0001	0.004
Current (mA)	Baseline	3.691 (2.669–4.801)	3.628 (2.602–4.612)	4.219 (2.776–6.189)
	Follow-up	3.953 (2.924–4.924)	3.913 (2.926–4.739)	4.595 (2.882–6.526)
	p (baseline vs. follow-up)	<0.0001	<0.0001	0.02
Change in impedance (%)		−5% (−10% to −1%)	−5% (−9% to 0%)	−4% (−10% to −1%)
	p (vs. 0)	<0.0001	<0.0001	0.005
Change in current (%)		4% (0–9%)	5% (0–9%)	4% (1–9%)
	p (vs. 0)	<0.0001	<0.0001	0.02

*Values are expressed as median (interquartile range). V, volts. mA, milliampere. Ω, Ohms*.

### Changes in Impedance and Current Over Time

**Figure 2:**
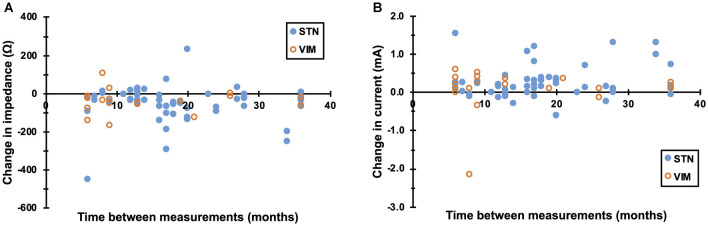
Rate of change of impedance and current. **(A)** Therapy impedance decreased at a rate of 27 ± 12 Ω/year (mean ± 2 standard errors; *p* < 0.0001). **(B)** Likewise, therapy current at a rate of 0.142 ± 0.063 mA/year (*p* < 0.0001). Values from STN and VIM electrodes are indicated by closed and open circles, respectively. Note that absolute values of impedance and current were used for statistical analysis.

The linear mixed model analysis demonstrated a significant relationship between time, therapy impedance, and therapy current. Impedance decreased at a rate of 27 ± 12 Ω/year (mean ± 2 standard errors; *p* < 0.0001). Current increased at a rate of 0.142 ± 0.063 mA/year (*p* < 0.0001). The relationship of time, current, and impedance is illustrated in Figure 2. There was no significant effect of electrode laterality on impedance (*p* = 0.96) or current (*p* = 0.56). Current was 0.998 ± 0.847 mA lower for electrodes targeting STN compared to electrodes targeting VIM (*p* = 0.02). Target was not significantly related to impedance.

## Discussion

### Impedance Drift With Constant Voltage

This is the first published study to examine DBS impedance over time when stimulation settings are kept constant. The longest interval without a change in stimulation settings was analyzed for each of 79 electrodes (60 in STN and 19 in VIM) in 44 patients (32 with PD and 12 with ET). Data collected within 6 months of electrode implantation were excluded due to early fluctuation in impedance (Lempka et al., 2009). Despite constant stimulation parameters, impedance still decreased by 27 ± 12 Ω/year, with a commensurate increase in the current of 0.142 ± 0.063 mA/year.

The rate of temporal impedance drift has previously been reported as −80 ± 8 Ω/year (Satzer et al., 2014). The lower rate in impedance decline observed in this study may indicate that changes in stimulation settings are partially responsible for impedance drift. Therapy voltage has been found to inversely correlate with electrode impedance, and contact activation has been associated with a more rapid decline in impedance (Satzer et al., 2014). This is consistent with data from animal studies showing a rapid decrease in impedance after acute stimulation, possibly due to oxidation at the brain-electrode interface (Lempka et al., 2009). Pulse stimulation has been used to restore lost signal-to-noise ratio in animal models (Johnson et al., 2005).

### Relationships With DBS Targets

While the target nucleus was not significantly related to impedance, the current was 0.998 ± 0.847 mA lower for electrodes targeting STN compared to that targeting VIM. Stimulation frequency was lower among STN electrodes, but it is not immediately obvious how lower frequency would result in the lower current. Prior research has found higher impedance for STN electrodes, and while the relationship between current and time has not been assessed in prior studies, Ohm’s law predicts that higher impedance would result in the lower current (Satzer et al., 2014). Anecdotally, ET-DBS patients tend to have higher voltage requirements, and higher voltage would correspond to higher current for VIM electrodes. While no significant relationship between target nucleus and voltage was observed in this study, the VIM subgroup was small, and the absence of a relationship between target, impedance, and voltage may simply reflect sample size.

### Study Limitations

One potential confounder is the reason for the absence of stimulation setting changes during the study period. Programming changes are often made when therapy is suboptimal, losing effectiveness, or associated with stimulation-induced side effects. The patients in this cohort did not experience significant clinical worsening during the study period, and therefore the findings of this analysis may not be generalizable to all patients undergoing DBS therapy.

Another caveat in comparing these findings to prior studies is the consideration of electrode vs. therapy impedance. Electrode impedance is measured for each contact at a standardized test voltage and is easily compared between subjects, whereas therapy impedance is measured between active contacts at the therapy voltage and has direct clinical relevance. Both types of impedance have been found to decrease over time (Satzer et al., 2014; Wong et al., 2018). The present study attempted to compromise between both types of impedance by recording therapy impedance exclusively from patients undergoing monopolar stimulation in periods without changes in stimulation setting.

This study was retrospective. The sample size was small, due to the strict inclusion criteria including an extended interval without a change in stimulation settings, although this trade-off was made to control for stimulation parameters, which varied in previous studies. Secondary analysis of data from larger prospective trials mandating fixed stimulation parameters could overcome these limitations.

### Implications for DBS Therapy

In clinical practice, programmed voltage is expected to increase over time to compensate for disease progression. This study suggests that increases in stimulation magnitude may account for some variation in impedance over time but do not fully explain impedance drift. Stimulation-independent impedance drift (and the corresponding increase in current) appears to be a very gradual and mild phenomenon but still serves as a motivator for constant-current programming. The clinical significance of this change requires further study.

## Data Availability Statement

The original contributions presented in the study are included in the article, further inquiries can be directed to the corresponding author.

## Ethics Statement

The studies involving human participants were reviewed and approved by the University of Chicago Institutional Review Board (IRB). The patients/participants provided their written informed consent to participate in this study.

## Author Contributions

TX: conception and design. TX, DS, and HY: analysis and interpretation. TX and MW: data collection. TX, DS, and HY: drafting the article. TX, DS, HY, MW, PW, MP, and MB: critical revision of the article. DS, HY, and MW: made equal contributions to the study. All authors contributed to the article and approved the submitted version.

## Conflict of Interest

The authors declare that the research was conducted in the absence of any commercial or financial relationships that could be construed as a potential conflict of interest.

## ABBREVIATIONS

DBS: deep brain stimulation
mA: milliampere
Ohms: Ω
V: volts
Hz: Hertz
μs: microseconds
STN: subthalamic nucleus
VIM: ventral intermediate nucleus of the thalamus.

## References

[B1] Cheung T. Nuno M. Hoffman M. Katz M. Kilbane C. Alterman R.. (2013). Longitudinal impedance variability in patients with chronically implanted DBS devices. Brain Stimul. 6, 746–751. 10.1016/j.brs.2013.03.010 23619246

[B2] Deuschl G. Raethjen J. Hellriegel H. Elble R. (2011). Treatment of patients with essential tremor. Lancet Neurol. 10, 148–161. 10.1016/S1474-4422(10)70322-7 21256454

[B3] Follett K. A. Weaver F. M. Stern M. Hur K. Harris C. L. Luo P.. (2010). Pallidal versus subthalamic deep-brain stimulation for Parkinson’s disease. N. Engl. J. Med. 362, 2077–2091. 10.1056/NEJMoa0907083 20519680

[B4] Hartmann C. J. Wojtecki L. Vesper J. Volkmann J. Groiss S. J. Schnitzler A.. (2015). Long-term evaluation of impedance levels and clinical development in subthalamic deep brain stimulation for Parkinson’s disease. Parkinsonism Relat. Disord. 21, 1247–1250. 10.1016/j.parkreldis.2015.07.019 26234953

[B5] Johnson M. D. Otto K. J. Kipke D. R. (2005). Repeated voltage biasing improves unit recordings by reducing resistive tissue impedances. IEEE Trans. Neural. Syst. Rehabil. Eng. 13, 160–165. 10.1109/TNSRE.2005.847373 16003894

[B6] Lempka S. F. Miocinovic S. Johnson M. D. Vitek J. L. McIntyre C. C. (2009). *in vivo* impedance spectroscopy of deep brain stimulation electrodes. J. Neural Eng. 6:046001. 10.1088/1741-2560/6/4/046001 19494421 PMC2861504

[B7] Okun M. S. Gallo B. V. Mandybur G. Jagid J. Foote K. D. Revilla F. J.. (2012). Subthalamic deep brain stimulation with a constant -current device in Parkinson’s disease: an open-label randomized controlled trial. Lancet Neurol. 11, 140–149. 10.1016/S1474-4422(11)70308-8 22239915

[B8] Satzer D. Lanctin D. Eberly L. E. Abosch A. (2014). Variation in deep brain simulation electrode impedance over years following electrode implantation. Stereotact. Funct. Neurosurg. 92, 94–102. 10.1159/000358014 24503709 PMC4531050

[B9] Satzer D. Mauer E. W. Lanctin D. Guan W. Abosch A. (2015). Anatomic correlates of deep brain stimulation electrode impedance. J. Neurol. Neurosurg. Psychiatry 86, 398–403. 10.1136/jnnp-2013-307284 24935985 PMC13349391

[B10] Vidailhet M. Vercueil L. Houeto J. L. Krystkowiak P. Benabid A.-L. Cornu P.. (2005). Bilateral deep brain stimulation of the globus pallidus in primary generalized dystonia. N. Engl. J. Med. 352, 459–467. 10.1056/NEJMoa042187 15689584

[B11] Weaver F. M. Follett K. Stern M. Hur K. Harris C. Marks W. J. Jr.. (2009). Bilateral deep brain stimulation vs best medical therapy for patients with advanced Parkinson disease: a randomized controlled trial. JAMA 301, 63–73. 10.1001/jama.2008.929 19126811 PMC2814800

[B12] Williams A. Gill S. Varma T. Jenkinson C. Quinn N. Mitchell R.. (2010). Deep brain stimulation plus best medical therapy versus best medical therapy alone for advanced Parkinson’s disease (PD SURG trial): a randomised, open-label trial. Lancet Neurol. 9, 581–591. 10.1016/S1474-4422(10)70093-4 20434403 PMC2874872

[B13] Wong J. Gunduz A. Shute J. Eisinger R. Cernera S. Ho K. W. D.. (2018). Longitudinal follow-up of impedance drift in deep brain stimulation cases. Tremor Other Hyperkinet. Mov. 8:542. 10.7916/D8M62XTC 29607241 PMC5876470

